# Tragedies of Technology: An Exploration of Such Narratives

**DOI:** 10.1007/s11948-026-00597-w

**Published:** 2026-05-14

**Authors:** Jan Grossarth, Armin Grunwald

**Affiliations:** 1 Department of Architecture and Energy Engineering, University of Applied Sciences Biberach, Karlstr. 6-11, 88400 Biberach an der Riß, Germany; 2 Institute of Technology Assessment and Systems Analysis (ITAS) at Karlsruhe Institute of Technology, Karlstr. 11, 76133 Karlsruhe, Germany

**Keywords:** Technology ethics, Technology assessment, Tragedy, Narratives, Hermeneutics

## Abstract

Technologies such as AI, autonomous drones, gene-editing tools, and climate engineering are profoundly transforming human life in the third decade of the 21st century. Experience of previous technological leaps points to ambivalent effects that at times may be considered “tragic.” This paper examines the relationship between technology and tragedy, contributing to the fields of technology ethics, technology assessment, and the philosophy of the human-environment relationship. The relationship between technology and tragedy is double-edged. On the one hand, technology can serve to eliminate tragedy, e.g. by ensuring safety by design, albeit never with guaranteed success. On the other hand, tragic experiences of technological consequences may result from the discrepancy between initial expectations and actual outcomes. A technological consequence is often framed as “tragic” if it was initially associated with high expectations of existential improvement (first necessary condition). Furthermore, a consequence can be labeled “tragic” in perspective only. Tragic is a personal or cultural narration, experiences that a technology’s promise of progress is at least partially reversed to produce the opposite (second necessary condition). As elaborated in this paper, tragic consequences can manifest in two ways: *First-order tragedy* refers to direct reversals of intended effects (e.g. a technology designed to alleviate hunger exacerbates it). *Second-order tragedy* involves gradual qualitative changes in life, such as diminished human agency or reduced participation – as if humankind were to become “part of a techno-economical system” and lose the freedom to shape life. This is the “tragic of the machine”. Ultimately, some “tragic technologies” narrations may also reflect the one-sided projection of experiences of tragedy onto technology.

## Highlights

The article provides a first systematic discussion on tragic tech consequences in the context of technology ethics and technology assessment

Elaborates key tragic elements

Differentiates between first- and second-order tragic technological consequences

Recommends discussing hermeneutic expansions of technology assessment

Discusses many examples from economic and technology history

## Introduction: “Tragedy” As a Category of Technological Modernity?

Tragedy has been a successful literary genre for at least two thousand five hundred years (Aylen, 2025). The term is an ancient category, but it is also vital as an expression of everyday-life diction: Many people can relate to the experience of “tragic” life stories, moments, turning points, loss of controllability. The category of the tragic reflects a basic feature of human existence, which always comes down to failure, illness, and death. A sense of the tragic in life connects people across time, class, and ethnic and cultural affiliations. This article explores the phenomenon of tragic experience in constellations involving technology.

The experience of tragedy is highly topical. In an era of disruptive technologies such as artificial intelligence or biotechnological revolutions such as CrisprCas9, the ancient tragedy of *Icarus* could acquire a new allegorical relevance. Icarus symbolizes the story of humankind, for whom a technical device not only serves as a tool enabling work to be done faster or more precisely, but also fundamentally extends humankind’s reach and scope of action (Wilkoszewska, 1986, p. 38). This gives rise to a changed perspective and a changed power relationship vis-à-vis the environment, but also vis-à-vis other people, animals e. g. It allows the technology’s owners or users to experience an expanded power – put in terms analogous to the story of Icarus, this contains elements of the dream of “ascending to the sun” (cf. Chap. 4.1.2; Grossarth & Grunwald, 2025). For others, however, this story can lead to a sense of helplessness and sadness. Icarus‘father Daedalus has to watch powerlessly as his son, having flown too close to the sun, plunges to his death despite his miraculous feathered wing.

There are numerous examples of “obtaining power” via technology. Others relate to imaginaries of the controllability of risks or dangers through technical means. Powerlessness in the face of imminent tragedy also characterizes the crash of *Germanwings* flight 9525 over the French Alps in 2015. With the promise of preventing dramas of the terrorist attacks of September 11, 2001, the European Union and other countries stipulated that airlines must equip cockpits with security locks that could not be opened from the outside in order to prevent aircraft hijackings. The possibility that the danger could come from pilots themselves was not taken into account: “This reinforcement of cockpit doors was motivated by security reasons, assuming that the threat to public safety came from outside the cockpit. (…) A potential security threat from inside the cockpit was not fully considered in either the initial phase or the period that followed, when the regulations were fine-tuned” (BEA, 2015, p. 93).

**Fig. 1:**
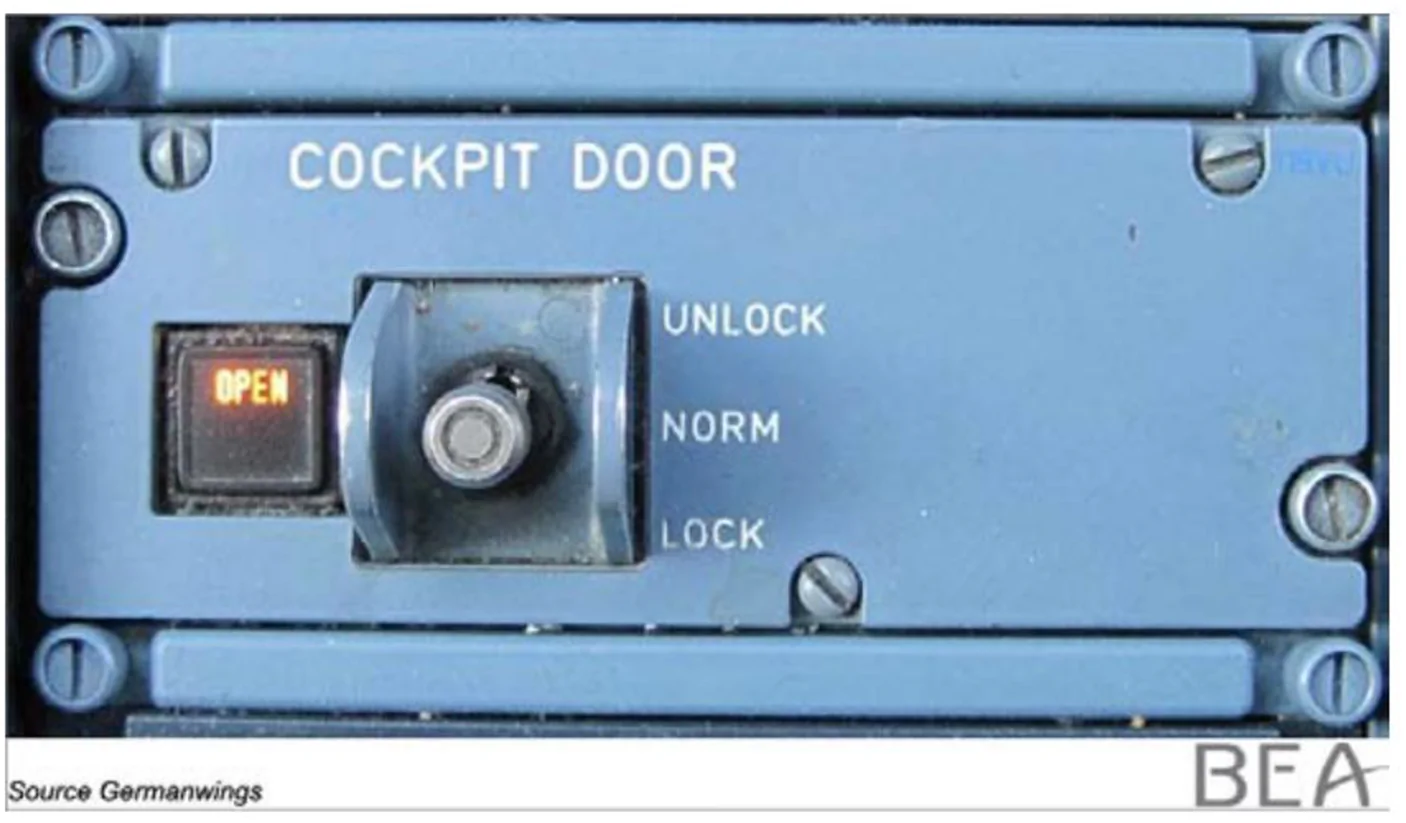
Toggle switch in the Germanwings aircraft (BEA 2015)

The crash of the Airbus A320-211 was caused by the co-pilot committing suicide. The *cockpit door lock system* (Fig. 1) enabled him to lock himself inside the cockpit on his own and deliberately crash the aircraft. The captain was outside the cockpit and could not re-enter. Utterly powerless, he was forced to stand by and let the tragedy happen. The understandable notion of using technical safety measures in an attempt to prevent future human suffering in fact led to new suffering in this case. The cockpit had “become a fortress” (Tagesspiegel, 2016) – a protective mechanism that cost 157 people their lives. A technology that was supposed to prevent mass fatalities made such a thing possible in the first place. Media coverage focused on the pilot’s mental illness, possible failings on the part of the airline and the failure of control mechanisms (von Heydendorff & Dreßing, 2016). The question of the event’s tragic dimension – not in relation to this act, but with regard to the history of technology and the political expectations of technological progress – was hardly raised. The main focus of the public debate was on identifying responsibilities: Could the disaster have been prevented by better administrative measures, tighter medical controls or stricter regulations? However, this perspective ignores the fact that the accident is an example of a deeper dimension of negative technological consequences that is rarely discussed in technology assessment: the tragic dialectic of the promise of safety and loss of control. At the same time, the example shows how difficult it would be to clearly define a “tragic technology”. Such a framing clearly depends on perspective. The pilot is hardly the tragic figure here; he was mentally ill and committed a crime under conditions of questionable culpability. However, his parents could be considered tragic subjects. Did they encourage their son to become a pilot despite his illness—to “rise high” in spite of everything? The developers of the door-locking system are also of interest. What promises were associated with its development and its recommendation to policymakers? Were they aware of possible unintended effects? The relatives of the victims possibly felt another kind of tragic experience – why was exactly this flight affected and not another one? One characteristic of the tragic is that, from a certain point onward, all possible actions lead to a calamitous outcome. In the case of the plane crash, this condition applies from the perspective of the co-pilot as well as the passengers and crew, but not from the perspective of the pilot, who could have intervened at any moment. These reflections suggest that tragedy is never objectively given, but is always experienced by someone. This experience is embedded in personal, cultural, and historical contexts.

Nevertheless, it may be useful to seek criteria for identifying such narratives. An experience of and reflection on tragedy can be shared and communicated intersubjectively (Slavin, 2016). Tragedy is perceived by an observer of an action. This article aims to clarify the extent to which the term “tragedy” describes something specific with regard to the consequences of technology by comparing it with established concepts such as “dilemma” or “ambivalence.” Our hypothesis – illustrated by the Germanwings story mentioned above – is that, in addition to the widespread expectation that technology can “eliminate tragedy”, there is also the possibility that it can create new tragedy, as experienced by observers.

This approach is new in the context of technology assessment and technology ethics while technology and tragedy have occasionally been discussed together in academic literature, though only as examples rather than systematically (see also Sect. 3 “The State of Research and Reflections on Technology and Tragedy”). Only few and weakly relevant examples can be found. The example of medical technology has been used to examine the tragic relationship between humans and technology (Verhey, 2002): medical technology may prolong life, but can – in the case of a pacemaker, which gives the heartbeat a uniform rhythm – “dull” physical sensation at the same time (Böhme, 2012). In the context of the “planetary age,” Trautsch has interpreted narratives of the tragic nature of technology (Trautsch, 2020, 2023, 2024). The “Promethean nature” of humans has been emphasized by citing the Anthropocene (Lawtoo, 2024). In technology assessment, technology phenomenology and technology ethics, however, the topic of tragedy has hardly been addressed to date (Sect. “The state of research and reflections on technology and tragedy”).

Our aim is – as far as possible – to lay down criteria for a better understanding of the phenomenon of tragic experiences with technology. This is intended to define the specific semantics of tragedy in the field of technology so that it can be used productively by disciplines that reflect on technology, such as STS, the philosophy of technology, the history of technology, technology assessment, and technology ethics. This involves answering questions about the criteria that define “tragic technological consequences,” about the different “depths” of an experience of tragic technological consequences, and about the specific characteristics of possible tragedy in the digital transformation. To this end, we will first study the connections between technology and tragedy by searching for examples in historical and current areas of technological consequences in order to collect material for systematic analysis (Sect. “Tragedy and Technology”). A brief look at the state of the research on the subject of technology and tragedy reveals that little attention has been paid to the phenomenon of tragedy so far, but that interest has grown recently, especially in phenomenology. In the main section “Systematic Thoughts About the Semantics of Tragic Technology” we develop a systematic approach to the category of tragedy in technology and the consequences of technology. The conclusion summarizes and offers an outlook in the form of ten theses.

## Tragedy and Technology

Technical innovations are shaping human life in the 21st century. They enable modern civilization by bringing about prosperity, mobility, and health in large parts of the world. At the same time, however, they harbor risks on a historically unprecedented scale, with considerable potential for tragedy. More than eight billion people – increasingly digitally interconnected and supplied with information – currently live on the planet, more than ten times as many as in pre-industrial times (Haub, 1995). The industrial mass production of food, goods, and medicines has increased the life expectancy of a person in Europe at birth from well below 30 years in 1750 to more than 70 years in 2020 (Torres, 2019, p. 2; Davenport, 2021). The unintended consequence of this tremendous success of technology is the massive overuse of the natural environment (see Grunwald 2009) and its considerable potential to bring about developments experienced as tragic, such as accelerated climate change and the depletion of planetary resources.

In the 1960s, the American non-fiction author Rachel Carson coined a dystopian narrative of the insecticide DDT’s harmful impact on the planet; this had the characteristics of a tragic narrative and shaped the American environmental movement (Lytle, 2007; Twidle, 2013; Grossarth, 2018). The “imagined tragedy may easily become a stark reality we all shall know”, as Carson pointed out (Carson 2000 [1962], p. 22). Decades before Carson, chemical-based agricultural technology had contributed another episode to the historical experience of the tragic. The invention of synthetic ammonia by the German chemists Haber and Bosch, which had a revolutionary impact on agriculture, led to consequences that have been perceived as tragic by many: though intended to help combat food shortages and hunger, the products were also used as a weapon during the First World War (Charles, 2011). Furthermore, they led to humanity’s dependence on energy-intensive, industrially produced nitrogen and altered nitrogen cycles in soils and waters (Smil, 1997, 2018).

These examples illustrate that it is precisely such successes of modern technology that can lead to interpretative approaches of tragedy. Even Stone Age inventions could be considered “tragic,” however. One prehistoric example is the flint knife: invented for the purposes of cutting up animals after a hunt in order to ensure a supply of food and people’s survival, it was also used as a weapon to kill people (Trautsch, 2020, p. 208). The sharp increase in what are known as diseases of civilization, which result from lives being prolonged by medical means, has been described as a tragedy (Verhey, 2002). An example from the world of building materials: Asbestos was long regarded as a miracle material for the construction industry and was used worldwide. As a result, however, hundreds of thousands of people have died of asbestosis, lung cancer and mesothelioma (Greenberg et al., 2002). One particularly tragic aspect of mesothelioma is that there is a latent period of several decades between the inhalation of asbestos fibers and the onset of the cancer, making a clear causal attribution – in other words, the attribution of responsibility – difficult.

Perhaps the most impressive example of “tragedy in success,” however, is man-made climate change (Gardiner, 2011). Its main cause is the consumption of fossil energies with the aim of bringing development, prosperity, and mobility to ever larger parts of the world’s population, i.e., to enable people to live a better life. Yet climate scientists expect around 14.5 million deaths to be attributable to climate change by 2050 (WEF, 2014), a price tag that certainly deserves to be called tragic.

**Fig. 2:**
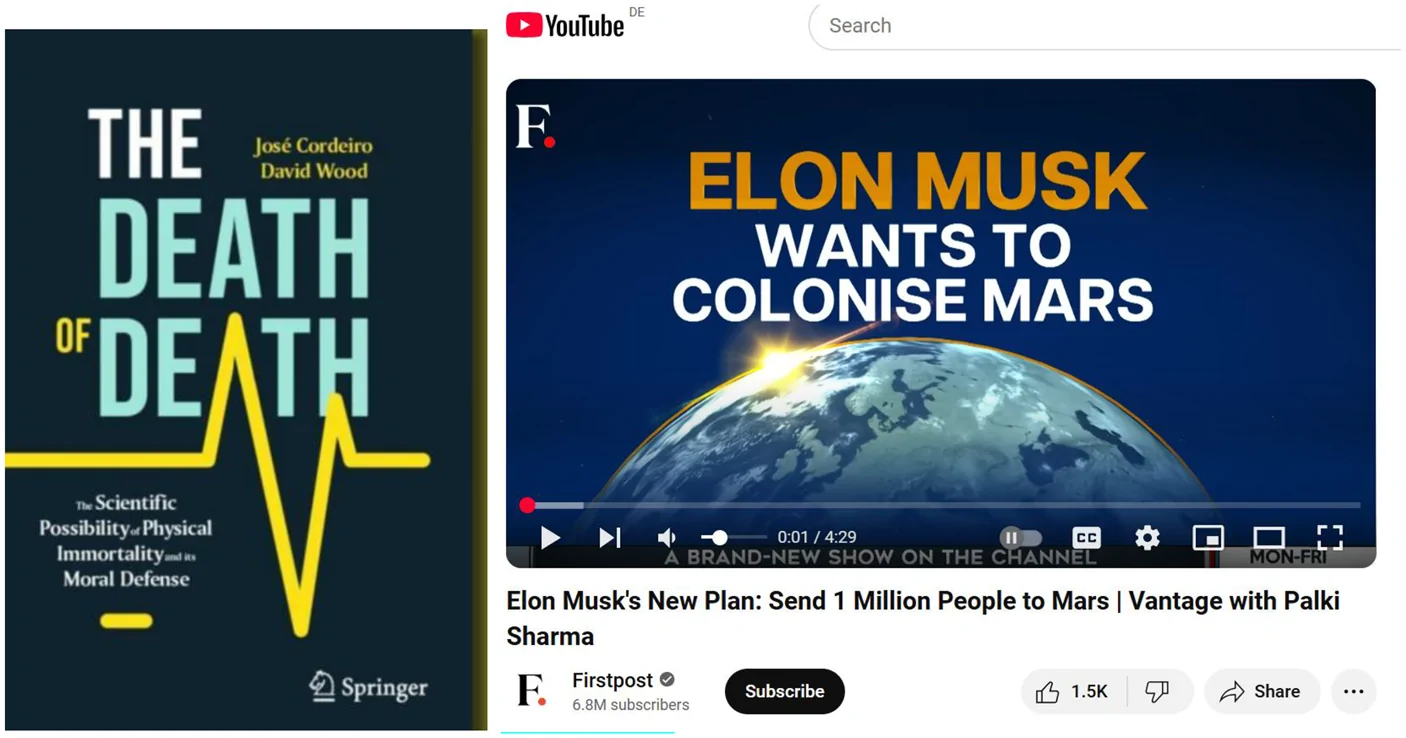
Leaving death and the earth behind – off to a better life? Book title and YouTube post

Technologies that promise salvation harbor particular potential for tragedy, as can be seen, for example, in the field of human enhancement through AI-based medicine (Szocik & Wójtowicz, 2019; Nyholm, 2024). Books with such bold titles as “The Death of Death” (Cordeiro & Wood, 2023) illustrate both the biotechnological ambitions and the potential for failure; Elon Musk’s Mars colonization project also has such qualities (Musk, 2017; Fig. 2). Early hopes that internet communication could have the power to foster democracy gave way to the realization that communication via social media – in the form of manipulation, fake news, hate speech, and filter bubbles – could actually pose a threat to democracy (Burton, 2023). Other technologies that are associated with the digital transformation and entail potentially tragic constellations include the smart generation of images and text in the form, for example, of *DeepSeek* or *ChatGPT*, whose success could lead to people losing precisely those core skills that make them human or becoming largely superfluous on the labor market. This example, too, illustrates the “tragic reversal.” The desire to make life easier by technological means leads to sociocultural disruptions. While these observations are covered by the established notion of the “ambivalence of technology” (e.g. Grunwald 2009), their experience or perception as “tragic” is a qualitatively specific aspect of this ambivalence (see Sect. “Systematic thoughts about the semantics of tragic technology”).

The idea of “eternal life through biotechnological progress” also calls to mind the jellyfish *Turritopsis dohrnii*. It is the only known multicellular organism with the ability to rejuvenate itself. Transferring its genes to human cells should, or so the vision goes, enable “eternal life” (Velasco-Muñoz et al., 2025). But what does this mean in terms of quality of life? If, as many philosophers have argued, human life owes its particularity, meaning, dignity, and value to its finiteness, which gives meaning to every moment of life (e.g., Heidegger; Krell, 1990), moving toward an infinite existence in this way could mean personal perceptions of a complete loss of these constitutive characteristics of being human. Achieving mankind’s dream of infinite life – explicitly denied to man in the Bible – would not mean embarking on a life full of “joys in abundance,” but rather the complete negation of culturally familiar meanings and of any value. A greater potential for tragedy is hardly conceivable.

## The State of Research and Reflections on Technology and Tragedy

The tragic in the history of technology has recently been described as a “structured constellation centered on rational action under conditions of epistemic limitation” (Schlaudt, 2025, pp. 1–5). The (aesthetic) category of the tragic foregrounded irreversibility and a “loss of prospective agency”, while a contrasting narration of technological progress as an “experiment” understood the trial and error of technology usage as “productive learning process” (ibid.). The ancient rooted category opposes the modern solution-oriented technological mindset (Carvalho, 2025). The concept of the tragic has also been made fruitful to expose the gap between digital processes and human lived experience, as in hopes for resonance and their disappointment in social media interaction (Sever, 2025; Lisenkova et al., 2025).

The one-sided interpretation of technological development as progress was already called into question in the second half of the 20th century (Chambers, 1958). Until then, any discussion of the semantics of progress was confined to the scientific-technical field; social or lifeworld concerns such as justice, happiness, or identity were not negotiated (Habermas, 2009). Nevertheless, even the “classical” philosophers of modernization and its dialectics of the second half of the 20th century, such as Habermas and Adorno, very rarely speak in terms of the tragic (cf. selected essays in Hanks, 2009, pp. 157–198).

The tragic structure of technological progress has been addressed in traditions of the philosophy of technology (Ellul, 1962; Dupuy, 2022; Hård & Jamison, 2013) as well as in analyses of technology narratives (Macnaghten et al., 2019). Our scrutiny of tragedy ties in with those works on the history and philosophy of technology, but extends the consideration of tragic experiences and perceptions of technology into the context of TA and ethics. As a result of major technological accidents perceived by many as tragedies such as Chernobyl in the 20th century, the ambivalence of technology has prompted a wealth of publications in these fields (e.g., Grunwald 2009; Van de Poel & Royakkers, 2023), as well as discourses on coping strategies such as the responsible and reflective use of technology or lifestyle-related sufficiency (Wiesing, 2020).

There has rarely been talk of tragedy in the ethics discourse (Schmidt, 2007), though it has been mentioned occasionally: one exemplary study draws on the concept of tragedy to challenge the way in which blame was attributed to individual engineers in the case of technical disasters such as the 2010 explosion of the Deepwater Horizon oil rig in the Gulf of Mexico (Coeckelbergh, 2012). In the 1980s, the philosopher of technology William W. Lowrance emphasized the dual role technology plays as both a problem and a solution with regard to risky technologies such as nuclear power plants: “I must emphasize that even while I stress the *essentially tragic nature of our awarenesses, decisions, and commitments*, I believe that we are in a great many ways better off than ever before” (Lowrance, 1986, pp. 145–150, emphasis JG/AG). Works from the 1970s that identified differences between tools and technology also merit attention. In the 1980s, Illich envisaged a transition from the “age of the tool” to an “age of systems” in which human action is to a certain extent completely subordinated to the evaluation categories of a “system” such as medicine, school or science (Illich, 1973, 2020; Samerski, 2018). Through machines, humans acquire historically unprecedented “exosomatic” power – power that increases the radius and strength of their own bodies to an extent never previously possible (Georgescu-Roegen 2019 [1982]; Mayumi, 2001). Industrial mining makes economic and technological development possible, yet it destroys landscapes (Francaviglia, 1997). Wind turbines contribute to the decarbonization of the energy industry, but in large numbers they threaten the aesthetic view on landscape. This ambivalence of technology is perceived as tragic in the name of socio-ecological sustainability.

Overarching gaps in research on technology and tragedy are evident in the following dimensions. *(1)* In the analytical dimension, there is a lack of criteria for systematically differentiating between the cases and contexts in which technological consequences are considered as “tragic” and in which not. (2) Previous work makes little or no reference to the rhetoric in *public discourse*: the semantics of tragedy has hardly been systematically placed in the context of public debates on technology. (3) Finally, the perspective of tragedy has yet to be integrated into or at least put in context of technology ethics and technology assessment. While the occurrence of unintended effects of technology and its fundamental ambivalence are well-established categories of reasoning about technology in Modernity (e.g., Beck 2014 [1986]), this paper adds substantially to this diagnosis. The focus on *tragic* experiences with and perceptions of technology and the technological advance shifts the attention to human observers and to people affected by asking the question for their interpretation of specific events or developments related with technology. Simultaneously, the narratives used in those observations and reflections are of interest in this context.

There has been virtually no talk of tragedy as yet in the application-oriented disciplines of technology assessment (TA) and technology ethics (Grunwald & Hillerbrand, 2021). This is surprising, given that weighing up opportunities and risks is at the heart of TA (Böschen et al., 2021). Exploring the connections between technology, impacts of technology, and tragedy, as this paper is doing, constitutes a new and unusual approach to technology assessment (TA). TA has been established in Western democracies since the 1970s. Initially, it was based – in line with the tradition of scientific rationality – on the idea of objectification, including in questions of evaluation, in full knowledge about consequences of technology, suggesting that full control in the sense of cybernetics and planning optimism (Camhis, 1978) would be possible to realize. The criticism that this approach may perpetuate an expectation of controllable technological consequences that does not reflect actual experiences of technical innovations was voiced early on (Wynne, 1975).

Indeed, this scientistic approach did not work, motivating TA’s turn to take limitations of knowledge, the uncertainty of prospective knowledge and the infiniteness of possible consequences of technology into account (Grunwald 2018). In addition, the participatory turn of technology assessment (Klüver, 2024) put the social dimension of values and interests in shaping and using technology more to the foreground. In these conceptual learning processes and changes, the notion of *responsibility* in decision-making over and use of technology became more important because of the uncertainties involved – which motivated, e.g., the emergence of Responsible Research and Innovation (RRI, cp. Owen & Pansera, 2019).

In spite of this opening processes with regard to low controllability of the technological advance over the last decades, the notion of tragedy hardly figures at all in the many studies of unintended technological consequences (Alberts, 1996; Harrison et al., 2007; Jago et al., 2024). In recent years, an approach has been developed – namely hermeneutic TA (Grunwald, 2014; Mehnert & Grunwald, 2024) – in which it would at least be possible to address the potentially tragic consequences of technology. In hermeneutic TA, narratives about technological futures are regarded as contemporary texts by contemporary authors with contemporary intentions rather than as statements about future events. These texts – or artistic objects – as contemporary products are accessible to hermeneutic interpretation and experience in the humanities. This approach thus also opens up perspectives of knowledge in the area of experience or fear of tragedy. This has yet to be systematically carried out in the form of case studies, however. Supplementary studies based on qualitative methods would need to explore possible experiences of the tragic (Francis, 2010). The category “tragic” can be experienced by “someone,” yet it hardly seems to be objectifiable and certainly cannot be delegated to “technical experts.” (Turner, 2001).

Accordingly, we do not aim at establishing an ontology of tragic technologies at the side of the objects but rather we look at the perceptions of technology as experienced or feared tragic. Furthermore, we want to shed light into the field of narratives used for describing experiences perceived as tragic. Thereby, we add a novel field to better understand perceptions of technology reaching from detailed events like the *Germanwings* case introduced at the top of this paper up to complex tragic reversals at the level of the *Anthropocene* like climate change. We aim at establishing helpful differentiations to support analysis and communication about such tragic experiences and perceptions. By explicitly addressing the dimension of tragic in technology, we also underpin existing doubts regarding the controllability of technology and the prevention of tragedies by means of technology.

Our analysis shall provide a novel contribution to the emerging field of hermeneutical TA (Mehnert & Grunwald, 2024). While this approach already includes the involvement of disciplines like cultural studies, hermeneutic philosophy, and discourse analysis, our analysis opens up new interdisciplinary opportunities for including linguistics, narratology, and the rich corpus of literature on the tragic and tragedies into the interpretation of new technologies.

## Systematic Thoughts About the Semantics of Tragic Technology

In this section, central aspects of the phenomenon that is the “tragic experience with technology” are identified and examined in depth with a view to precisely defining two necessary conditions for such a framing.

### Aspects of Tragedy

Experience of the tragic never results from technology alone, but from the interplay with human expectations and experiences. People “process” experiences by creating meaningful narratives (Brown et al., 2008). Whether the use of the adjective “tragic” will be perceived as appropriate by a particular individual or in historiography will depend on complex contexts. Such contexts are biographical, motivational, historical, or atmospheric, or are based on analogies to history and literature (see box).

The following five criteria for framing an event as “tragedy” are highlighted in this section: ambiguous motives for action (1), tragic constellations instead of individual guilt and hubris (2), the existential dimension of the intended consequences of technology (3), the moment when a desired consequence is tragically reversed to become the opposite (4), and pity and fear as emotional reactions of the observers (5).

**Table:** 

**Constellations and Figurations**	
The concepts of *constellation* and *figuration* can be helpful for clarifying the complex multi-causality in the emergence of tragedy. The concept of constellation, which is commonly used in sociology, refers to the interaction of various historical, social, political, and technical factors which, in their specific combination, enable or shape a certain development (Heidegren, 2024). A constellation is also shaped by a specific era’s technical possibilities and cultural perspectives. It is such dynamic constellations that cause technological consequences to be interpreted as “tragic.” The concept of figuration was coined by the cultural sociologist Norbert Elias (Elias 2018 [1992]). Regarding groups, people, and technical possibilities as interdependent, this concept focuses on the interdependence of actors within structures. In this perspective, technology does not appear as an isolated artifact, but as part of a network of relationships in which people are both subjects acting in their own right and actors influenced by technology. In these figurations, tragedy often arises where the scope for action of individuals and collectives is unintentionally restricted by technological developments. Such interactions are also difficult to predict. The Germanwings crash is an example of this, given in the introduction.	

#### Unavoidable Actions, Ambiguous Motives

An assumption of incomplete knowledge about the consequences of the protagonists’ actions is central to tragic narratives (for the following explanations, see Trautsch 2020, pp. 187–200). The consequences of technology cannot be fully foreseen, yet action often has to be taken nonetheless. Individual responsibility is often not clearly attributable. The hero of Greek tragedy appears neither completely guilty nor completely innocent of the course of tragic events (Watts, 2021; Schimmenti, 2022). Neither fate determined by the gods nor the hero’s individual free choice alone are responsible for the tragic event. In Shakespeare’s royal dramas such as Hamlet, not only do the modes of heroes and anti-heroes stand in opposition to each other as “power” and “love;” but above all one is faced with ambivalent motives for “good”: Even the actions of Hamlet or Ophelia, who come together despite the resistance of the Queen, are not clearly motivated per se (Wilkoszewska, 1986, pp. 34–39). Tragedy thus eludes any clear causal attributions of guilt. It is based on the realization that people often – and especially in borderline situations – have to act in a state of uncertainty about their own motives and the consequences of their actions. Affects, gaps in knowledge and a lack of knowledge about the future shape their decisions. Humans cannot reliably assess the extent of their control over the course of events, so they overestimate it. Nevertheless, humans are forced to take (technical) action: due to scarcity, threats, or natural hazards, or simply because of the human desire to deploy their knowledge and skills (Meier, 1978).

#### Beyond Hubris: Multiple Perspectives Embedded in Constellations of Hope, Success and Failure

Precisely because technical innovation is “essential for survival” in the light of the human experience of scarcity, and because there is also uncertainty about future application constellations, even the boldest technical innovation cannot be lightly discredited as “hubris.” Consequently, any tragic consequences can hardly be interpreted as a “punishment” for pride and overconfidence (even not in secular contexts). As far as the motives for action are concerned, a tragic consequence of technology therefore differs from the tragic epic hero, whose actions are usually characterized by “tragic pride […], arrogance, overconfidence” (Castelli, 2022, pp. 61–65). Hubris would involve the frivolous *use* of technology. In technological modernity, however, tragedy is not necessarily nor primarily the result of hubris (of a single “hero”); rather the tragic consequences of technology can result from the best arguments (cf. the *Germanwings* and other examples in this paper). In contrast to literary tragedy, the mantle of the hero is, so to speak, spread across several shoulders.

**Fig. 3:**
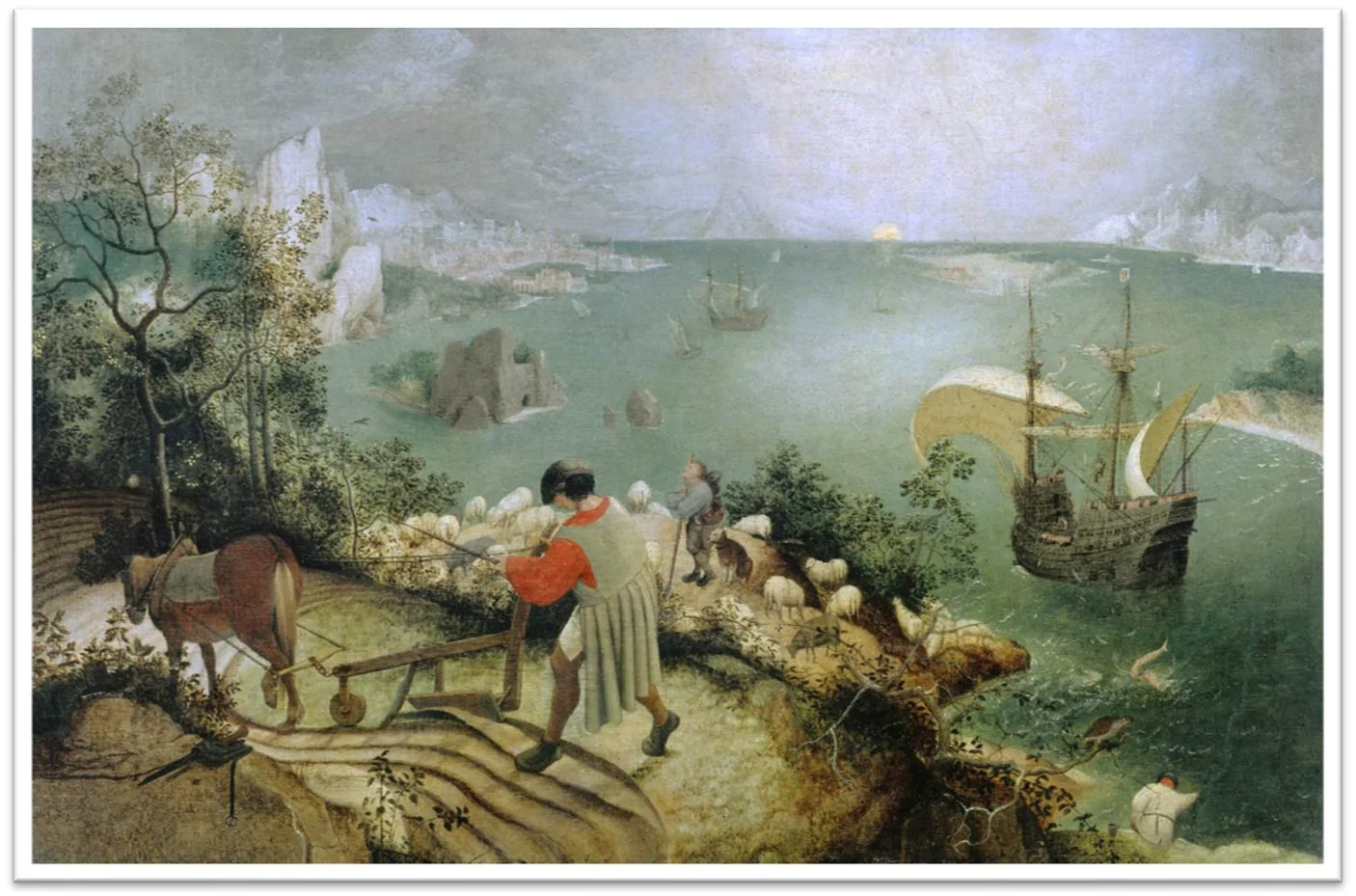
Landscape with the Fall of Icarus, Bruegel the elder (attributed), royal museums of Fine arts, Brussels

Such heroes are rarely in the “limelight,” unlike the protagonists of a royal drama. The “modern Icaruses” (or Deadaluses) do not necessarily attract attention; many remain largely unknown in the background (Elon Musk would certainly be an exception). An illuminating example of this realization can already be found in Bruegel’s early modern painting “Landscape with the Fall of Icarus” (dated around 1555) (Fig. 3).

The figure of Icarus – often interpreted as a symbol of hubris – is small enough to be easily overlooked in the painting, and is barely larger than one of the sheep. Meanwhile, the foreground is dominated by the plowing farmer, a shepherd, and merchant ships. They seem to be going about their business unmoved, as if the fall of Icarus warranted neither attention nor significance. The tragedy here is ironically magnified: it is not even recognized: “None of those present, except the tragic flier, participate in his accident or share his tragedy” (Wilkoszewska, 1986, p. 29). This way of looking at it can also be applied to the context of technological tragedy: the downfall of exposed technological heroes may be a question of individual fate, yet no general epochal insights follow from it. It remains historically irrelevant and has no consequences in terms of technology ethics. At the center of Brueghel’s humanistic view is not the spectacular fall, but the continuation of useful everyday activities such as plowing and sailing around the world – the technical accomplishments whose effects are proving to be highly advantageous for now. Tragedy here does not appear as a moral warning against “hubris,” but rather as a phenomenon that daily accompanies man, whose actions are inextricably ambivalent in a complex and opaque domain characterized by knowledge, uncertainty, and an acute need for action.

Perhaps the focus on Icarus is also misleading. Might it not rather be his father, Daedalus, who should be seen as the “tragic technician” in a constellation of need and good arguments for his technological development? He is the engineer, the developer of the flying device. He designs it for the purpose of escaping imprisonment on the island of Crete. Icarus, by contrast, flies too high for pure pleasure and, out of recklessness, plunges to his death. Yet it is Daedalus who experiences the truly tragic consequence as the powerless observer: he is the on reflection, how the motif of gaining freedom is reversed into the irretrievable loss of his son. But to put it clear here: A feature of the tragic is also, that the intentionality behind development is never entirely transparent. Beyond an explicit goal (here, freedom), there may be other driving forces—technical virtuosity, the exercise of control, external demands, curiosity, even mere boredom, or motives that cannot be clearly identified at all. Accounts of tragic experience tend to dissolve straightforward chains of causality and resist clear assignments of cause and effect (ch. 4.1.1).

#### A Multilayered Tableau of Motives, Circumstances, and Technical Aspects – and the Existential Dimension of Technology Promises

Technologies framed as tragic are often those that are associated with an expectation that they will existentially improve human living conditions yet whose application initiates or enables processes that run counter to this original value-based intention. Their consequences may not only contradict the intended goal, but also reverse it – in some cases even with fatal consequences (Barbour, 1983). Consequences of technology read as tragic therefore imply existential negative effects, especially in connection with technologies that:

- are designed to *save lives,*
- are intended to *improve the life of humankind as a whole,*
- are associated with hopes of increased *peace and freedom*,
- or entail an expectation that “evil” – in the form of *terror, violence or injustice –* will be fundamentally curbed.

A main focus is therefore on technologies that aim to “improve the world”. Precisely because they promise to fundamentally humanize life, their potential for downfall lead to understandings as tragic: Their success stories cannot be decoupled from the possibility that they will take a tragic turn under certain conditions. In this sense, the climate engineering approach to tackling climate change has been described in the media as having the potential for future tragedy, as it relies on large-scale technological solutions such as “solar sails” or weather manipulation for adaptation rather than on avoiding climate-damaging emissions (Buck, 2013). And a very different additional example: Western development policy for Africa, which was intended to improve living conditions but resulted in increasing dependency and over-indebtedness, has also been described as a tragedy (Adeboye, 1997).

If no claim is made that a particular technology will help resolve existential problems – in how far might it make sense to talk of tragedy? A falling roof tile that kills someone in the street is an interesting example. On the one side, it might be seen as something like a “great misfortune”, an unlikely accident, or a terrible fate. but certainly not tragic. That might be valid, if people do not put the technology of roof tiles into the context of promises of eliminating fatal accidents. Otherwise, roof tiles are, of course, intended to enhance human living conditions by shielding against existential threats such as storms, hail, and intense rainfall.

There are, moreover, additional interpretive spaces of the tragic. Even the case of a boulder falling onto a hiker in the mountains may be interpreted as tragic. Although this has nothing to do with technology, it points to a particular understanding of life and its incalculable finitude. Various contexts shape the interpretation: What brought the walker to that very place at that very moment? What would have happened had he left his home earlier, later, or not at all? A multilayered tableau of information about motives, circumstances, and technical aspects underpins interpretations of technology as tragic—never technology and the intentions behind its *development* alone. The example of the roof tile illustrates this point paradigmatically.

These reflections suggest that the tragic should be understood less as an objective property of events or technologies than as a culturally mediated mode of interpretation. The attribution of tragedy is highly presupposition-laden: it resides in the eye of the beholder and may well presuppose a literary education shaped by the tradition of classical tragedy. This background continues to inform even everyday linguistic usages, such as the phrase “tragic car accident.” In the improbable conjunction of timing, fatal outcome, and opaque motives for driving, a dimension of tragedy is often assumed—even if it cannot be clearly demonstrated or analytically differentiated. The perception of tragedy thus emerges from a complex interpretive framework rather than from the event itself. One may therefore assume the formative influence of a humanistic Western literary and philosophical tradition in shaping such interpretations. It is therefore debated for decades, whether Chinese traditions of interpretation display a similar structural disposition toward tragic narration (Chen & Qi, 2020; Zhao, 2014). At this point, new perspectives for hermeneutic technology assessment appear.

#### The Tragic Reversal

Any attempt to formulate “criteria” of the tragic remains plausible only within the interpretive horizons described above. One “criterion” for a technology tragedy is when an intended value is reversed and produces the opposite effect. Tragedy framings manifests itself in moments when something that was intended as progress or liberation becomes a source of suffering and entrapment. Structural parallels of such a reversal of salvation into disaster can be found in the dialectic of enlightenment, motivated by the industrialized genocide perpetrated in the Shoah, 9): “the wholly enlightened earth is radiant with triumphant calamity.” This reversal is often associated with the experience of “getting caught up in” the structures of power – be it the political power of states, the technical self-empowerment of the individual, or the systemic dynamics of technological development (Paster, 2002). As in the myth of Daedalus and Icarus, the fate of the tragic hero is inextricably linked to power and its ambivalence. Here, the “ability to fly” symbolizes not only technical progress, but also the allure of transcending oneself. Despite the warnings, Icarus flies ever higher, intoxicated by success, until he crashes. This moment of reversal is characteristic of literary tragedy: The flight of fancy is followed by a fall, hubris is followed by nemesis. Yet the experience of flying – of soaring heights – Is likewise a literary figure that frequently precedes the tragic fall (Grossarth & Grunwald, 2025).

Moments of reversal can be identified in the history of technological experience. All of them constitute examples of technologies whose history of effects can be narrated as tragic:

- The *atomic bomb*, originally intended as a means of ending war, marked the beginning of a global era of nuclear threat (Hiroshima; Farrell, 1995).
- *Nuclear energy*, a symbol of technological sovereignty, revealed its destructive potential at Chernobyl (Jasanoff & Kim, 2009).
- *Industrialization*, which brought prosperity, is also the cause of the climate crisis, which is now creating existential threats (summer droughts, extreme weather).
- Mass housing built with steel and concrete, which is associated with high greenhouse gas emissions, also serves to reduce social housing shortages (Grossarth, 2025) – security is achieved, but new insecurity is created.
- The first coal-powered steam locomotives were not used for tourism or pleasure. They connected poor rural regions to the industrially up-and-coming cities. They led to emancipation, i.e., to *gains in freedom* for large sections of the population. The consequences of climate change, on the other hand, entail new forms of a *lack of freedom –* whether for those forced to leave devastated regions of the southern Mediterranean or southern US states such as Arizona, or for future generations (Ferretti, 2023).

The Greek word *metabolē* (μεταβολή) refers to such a “change” or “reversal” – a sudden shift from progress to catastrophe, from hope to disaster. τὰ ἐναντία refers to a radical change to an opposite state. These terms articulate the core of the “tragic experience” (Trautsch, 2020, pp. 76–77). The myth of Prometheus is also a paradigmatic example of tragic entanglement with technology (Lawtoo, 2024): his gifts of fire and the art of metalworking were supposed to emancipate mankind. Yet it is precisely these technologies that are turned against him: Zeus has the demigod Hephaestus forge the chain that binds the benefactor Prometheus. Technology, a symbol of liberation, suddenly mutates into an instrument of oppression (Trautsch, 2020, 210ff.).

#### Pity, Fear and Sudden Fright as Emotional Reactions

Yet the tragedy of technology is truly plausible only as an interpretation of an inner experience. It is the first-person perspective that proves decisive in determining whether such an interpretation arises. The *Germanwings* example at the outset illustrated this point. If the pilot himself did not construe his “fate” as tragic—and if most observers of the crime do not do so either—the situation may nonetheless appear differently from the perspective of a father or a mother, or in light of their respective “fates” (Sect. “Introduction: “Tragedy” As a Category of Technological Modernity?”). The effect of tragic stories is a litmus test of their plausibility. The initial reaction is a kind of “startled awakening” – a very abrupt realization of a tragic reversal and a sense of being helplessly at the mercy of others. Furthermore, as Aristotle noted, there must be *pity* (for the hero) and *fear* (that it could happen to oneself) (Munteanu, 2011; Konstan, 2005, pp. 14–16). These criteria still appear suitable for classifying narratives about technological tragedies nowadays. Does the *Germanwings* crash trigger something like pity and fear in the onlooker? On which? Does the insolvency of an inventor whose invention made him a fast-rising and successful “innovator” just ten years ago, but who has now been “overtaken” and rendered obsolete by an even more innovative innovator, trigger pity and fear? If so, this story of “loss of agency” through “exemplary (entrepreneurial) commitment” would be a tragedy.

### Necessary Conditions for Speaking of the Tragic

**Fig. 4:**
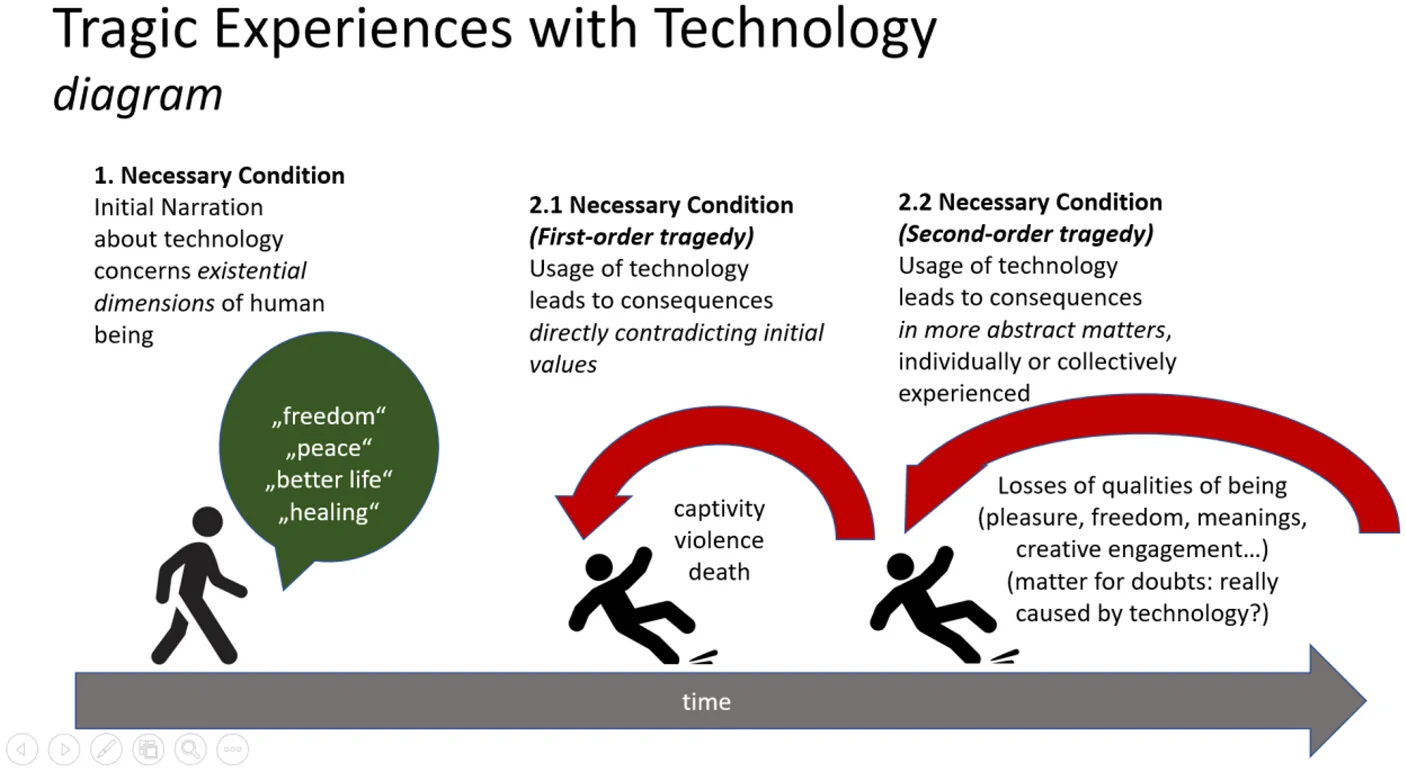
A diagram of the first- and second-order tragic experience of technology

We will now discuss in more depth two “necessary” conditions for the meaningful use of the words “tragic” or “tragedy” on the basis of the above considerations (Fig. 4). Both conditions must be fulfilled in order to rationally speak of tragic technology. We will also differentiate between tragic of first and second order.

1. We see the existence of an initial technology narrative with an existential dimension as a *necessary condition* to justify talk of tragic technological consequences. Whether this narrative was formulated and disseminated by inventors, developers, the media, industry, or other actors or institutions is irrelevant. Independently of the inherently relative and perspective-dependent narrative structure of the tragic, this condition can be assumed as necessary in order to rationally speak of tragedy. Examples of “technology narratives with an existential dimension” would be promises or hopes of more freedom, a better life for humanity, a cure for disease (or even death), an end to hunger or the securing of peace.
2. We consider it a *second necessary condition* if a *tragic effect* occurs immediately or after some period of time (minutes, hours, years, decades or centuries). The presence of a tragic effect (of the first or second order) therefore constitutes a second necessary condition for speaking rationally of the tragic.
  - *The tragic effect of the first order* is the *reversal* of events from the “good intention” to its opposite. The technological possibility itself serves only or also to destroy the values or to thwart the realization of the values for the sake of which it was brought into the world. The crash of the *Germanwings* plane – leading to hundreds of deaths – was an extreme event of this kind. The freedom to travel was perverted into unavoidable death.
  - We see the phenomenon of negatively experienced qualitative changes in life circumstances as a *second-order tragic effect*. History does not provide (such easily) objectively evidence as it does for first-order consequences. This is why they are also subject to conflicting interpretations and rarely consensual. They can be witnessed, lamented, and described by people. These effects concern factors such as changes in language habits or scope for action, socially accepted lifestyles, ways of working, the social legitimacy of leisure time or forms of expression, a disruption of meanings – qualitatively ascertainable factors. Their existence, gravitas or technology-related causality is by far not as clear as in the case of first-order technological consequences. To cite Hans Blumenberg, one could say that metaphors are needed to describe them – such as a loss of “depth,” “brightness” or “breadth” of the existence (Blumenberg, 1971). Those metaphors are linked to”background ideas” of the “good life”.

A critical point needs to be added regarding second-order effects. The possibility that people may *project* the tragedy of their general life experience *onto technology in a monocausal manner* must be raised here. Statements such as “Robots are taking away our jobs” (instead of: “Managers who decide to use these robots in this way”) or “My child suffers from ADHD because of tablets” (instead of: “because we gave him the tablet too early”; or “because we don’t have the energy to extensively engage with him”) or “I am ill because of industrially processed ready meals” (instead of: “because of my one-sided dietary choices to eat such meals”) would be examples of this. In this context, we could speak of technology-related discourse narratives.

Technology and tragedy appear to be in a double-sided relationship: on the one hand, technology is suitable for “eliminating tragedy,” albeit without any fundamental prospect of success and indeed even entailing the possibility of new tragedy. If the mere intention of the “owners” (users) of the technology changes, the purposes of its deployment can also change. Similarly, just as the course of history (cf. Koselleck, 1982; Zammito, 2004; Rosa, 2020) is understood as unavailable or beyond full human planning and calculability, the future “tragic effects” of technological innovations should likewise be regarded as unavailable.

## Conclusion: Ten Theses on the Tragic Consequences of Technology

In conclusion, the above thoughts can be condensed into the following ten theses:

1. Tragedy is an element of the *human condition* that addresses people’s existential situations and experiences. Many characters, dramas, and stories have explored and reflected on tragic experiences of the human condition. But interpretations of events as tragic rely on cultural contexts, education, literacy – they are *aesthetically* as made by observers of actions and their consequences.
2. In the modern age, and in the Anthropocene at the latest, technical thinking has *become dominant* in many areas. This applies in particular to problem-solving approaches. The moment a problem arises, there is a reflexive call for a technical solution or for technology to contribute to a solution, the typical patterns of thought being “problem and solution” and “cause and controllable effect.” Tragedy narrations contrast this illusion of controllability.
3. There is an irresolvable conflict between technical thinking and the need to address the possibility of tragedy. On the one hand, this concerns human relationship with the world: While tragedy relates to events that have already happened or are feared – a situation/event is perceived as tragic – technical thinking is oriented toward intervention and control to achieve a particular purpose.
4. On the other hand, technical thinking and problem solving promise to reduce or, ideally, completely prevent the occurrence of tragic situations by calculating and controlling the consequences of actions. Progress in this sense is regarded as the increasing elimination of the tragic in favor of the controllable. This is understandable from a certain point of view, as tragic events or situations are associated with suffering and misfortune. In many cases, the technological society has indeed produced successful strategies to reduce tragic constellations (infant mortality, world nutrition, access to information …) *However, in an attempt to eliminate constellations perceived as tragic, new “technology tragedies” are constantly being created*, for example, when well-founded technical measures produce unexpected consequences entailing their own tragedy, or when intended gains in control and freedom through technological progress are transformed into new dependencies and constraints.
5. Powerlessness becomes apparent in tragic constellations: events defy expectations of “cool-headed rationality” and even the best predictive and control knowledge. Things turn out differently than expected, and in some cases differently on an existential level (Fig. 5). The literature on tragedy regards this as a constitutive, i.e., non-eliminable aspect of being human. However, when technical thinking attempts precisely such elimination, profound questions about human beings, about being human and about human self-understanding come to the fore – this is a challenge to as well as an opportunity for hermeneutic technology assessment.

**Fig. 5:**
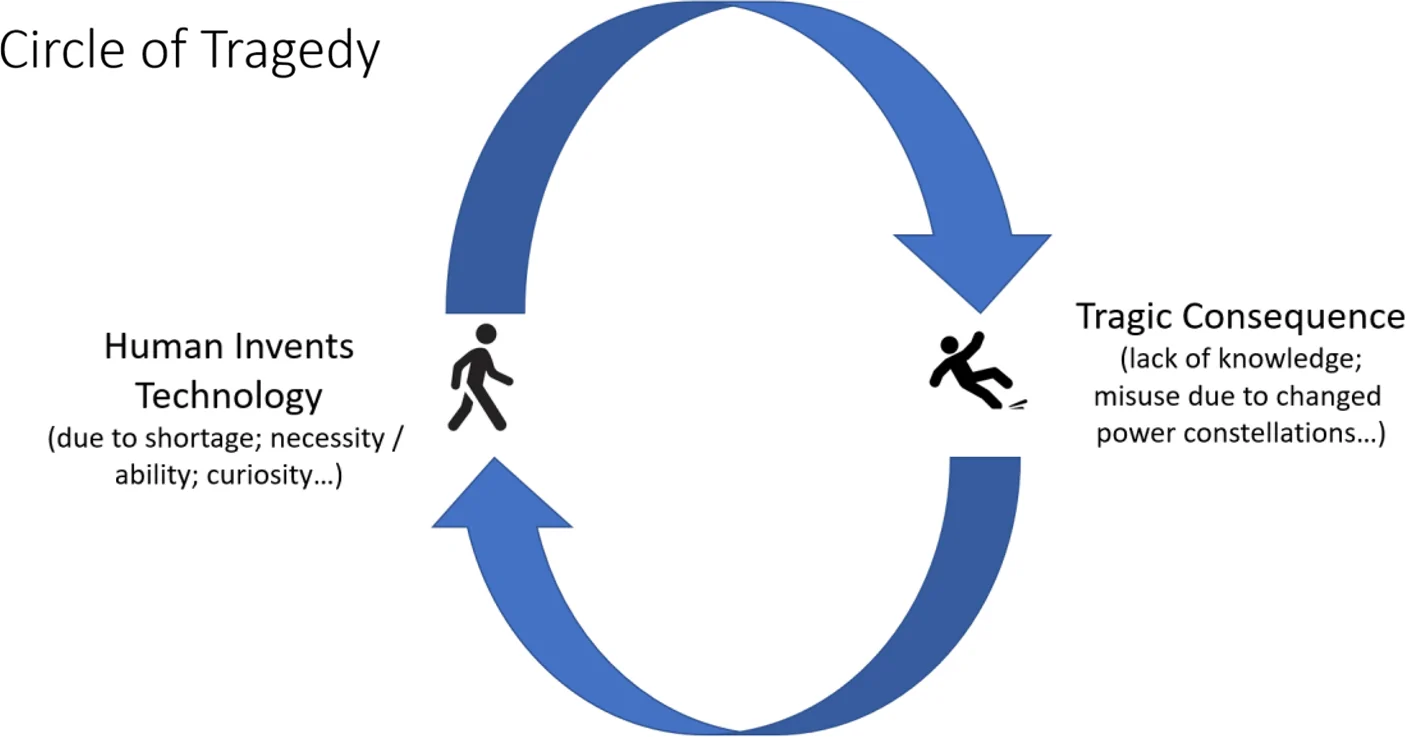
The cycle of tragic technical innovation
6. By highlighting the unavailability of unintended technological consequences and their potential existential “tragic outcomes”, a hermeneutically expanded technology assessment assigns ethics the task of keeping expectations of control, risk management, and security within the bounds of what is realistically possible.
7. As a conceptual distinction in analyzing technological tragedy we suggested *first-order* and *second-order tragedies*: while all tragedies of technology possess an existential dimension, first-order tragedies involve the destruction of concrete underlying values and are therefore open to deliberative negotiation within pluralistic societies, whereas second-order tragedies concern disruptions of broader structures of meaning. Although less accessible to deliberative resolution, they should nevertheless be acknowledged in public discourse due to the profound uncertainties they reveal.
8. In the modern age, accepting powerlessness is often seen as backward or pre-modern, e.g., in relation to our ultimate powerlessness in the face of death. Seen in this light, the *feeling of powerlessness and experiences of tragedy* are *drivers of technological progress –* which itself, however, does not necessarily lead us out of powerlessness.
9. In the modern age, this area of conflict is often addressed and communicated by the media, experts, lobbyists, and politicians, though without their being able to shrug off superficial expectations of tragedy being overcome through technology (cf. Sect. “Systematic thoughts about the semantics of tragic technology”).
10. The discourse on tragedy is on the decline amid our digital consumer culture and communication. This is in itself a tragedy on the meta level because the markets for tragedy narratives and reflection are disappearing. Digital media lead to a de facto compulsion to disambiguate. Belief-like positions, for example, on nuclear energy, are replacing the deliberative search for good paths. The scope for publicly addressing the tragedy of technology is dwindling.

To summarize, this also reveals two misconceptions about technology: firstly, the widespread misunderstanding that progress can eliminate tragedy. This assumption overloads technology with an unfulfillable expectation. Secondly, the belief that technical progress can unilaterally resolve dilemmas. In fact, technology is not only unable to eliminate tragedy, but often triggers new tragic developments in many different ways.

**Fig. 6:**
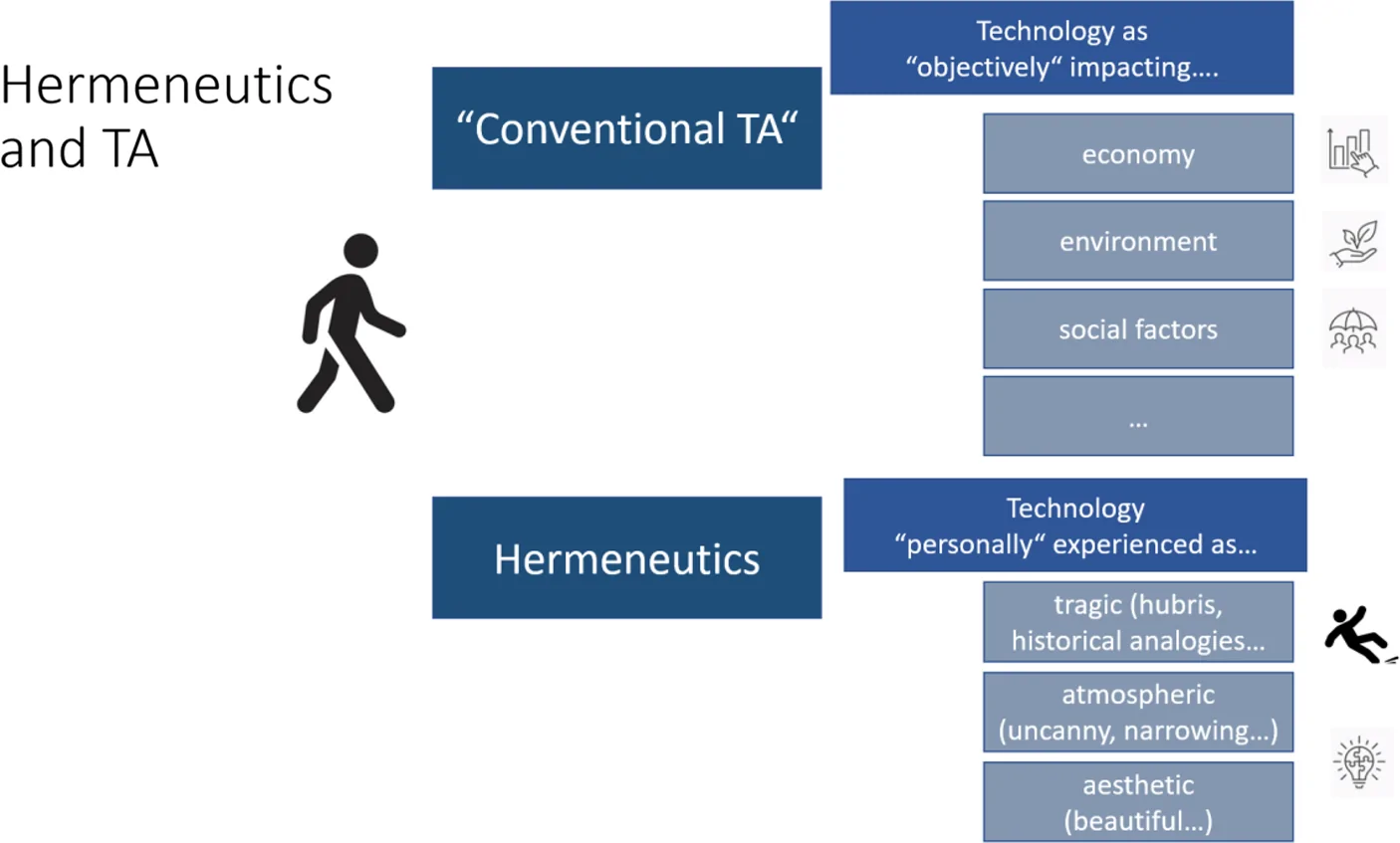
Hermeneutic expansion of TA to include experiences or expectations of tragedy

The use of technology is always embedded within application contexts that cannot be fully predicted and are shaped by political and economic power structures. This ultimately makes the consequences incalculable. As far as methodology is concerned, this makes it recommendable to also include personal and collective memories in the impact assessment. Biographical and historical memory can be explored hermeneutically (Fig. 6). It could usefully supplement existing methods of technology assessment (TA) – for example, through text, image, or interview analyses (Janich, 2024). An in-depth discussion of methods has yet to be conducted.

## Data Availability

No datasets were generated or analysed during the current study.
